# SPIN1 accelerates tumorigenesis and confers radioresistance in non-small cell lung cancer by orchestrating the FOXO3a/FOXM1 axis

**DOI:** 10.1038/s41419-024-07225-0

**Published:** 2024-11-15

**Authors:** Min Zhong, Zhi Fang, Juntao Zou, Xiao Chen, Zezhi Qiu, Ling Zhou, Yi Le, Zhen Chen, Yanyu Liao, Fengting Nie, Xianpin Wei, Jinbo Zhan, Jianping Xiong, Xiaojun Xiang, Ziling Fang

**Affiliations:** 1 Department of Oncology, the First Affiliated Hospital of Nanchang University, Nanchang, Jiangxi province PR China; 2 Jiangxi Key Laboratory for Individualized Cancer Therapy, Nanchang, Jiangxi province PR China; 3 Department of Respiratory, the First Affiliated Hospital of Nanchang University, Nanchang, Jiangxi province PR China

**Keywords:** Non-small-cell lung cancer, Mechanisms of disease

## Abstract

Despite the importance of radiation therapy as a nonsurgical treatment for non-small cell lung cancer (NSCLC), radiation resistance has always been a concern because of poor patient response and outcomes. Therefore, it is crucial to identify novel targets to increase the effectiveness of radiotherapy and investigate the mechanisms underlying radioresistance. Previously, we demonstrated that Spindlin 1 (SPIN1) was related to tumour initiation and progression. In this study, we found that SPIN1 expression was higher in NSCLC tissues and cell lines than in the corresponding controls. SPIN1 overexpression in NSCLC patients was closely correlated with disease progression and poor prognosis. Functionally, SPIN1 depletion inhibited cell proliferation, decreased the percentage of cells in the G2/M phase and suppressed cell migration and invasion. Moreover, SPIN1 knockdown decreased the clonogenic capacity, impaired double-strand break (DSB) repair and increased NSCLC radiosensitivity. Mechanistically, forkhead box M1 (FOXM1) was identified as a key downstream effector of SPIN1 in NSCLC cells. Furthermore, SPIN1 was found to facilitate MDM2-mediated FOXO3a ubiquitination and degradation, leading to FOXM1 upregulation. Moreover, restoration of FOXM1 expression markedly abolished the inhibitory effects and increased radiosensitivity induced by SPIN1 depletion. These results indicate that the SPIN1-MDM2-FOXO3a/FOXM1 signalling axis is essential for NSCLC progression and radioresistance and could serve as a therapeutic target for increasing radiotherapy efficacy.

## Introduction

Lung cancer is the leading cause of cancer-related mortality worldwide, and non-small cell lung cancer (NSCLC) accounts for more than 85% of lung cancer cases[1, 2]. Despite the tremendous progress in NSCLC treatment, the overall survival of NSCLC patients is still unfavourable [3]. Radioresistance is considered a key contributor to local recurrence and distant metastasis in NSCLC patients [4]. Therefore, elucidating the molecular mechanism underlying cancer radioresistance and identifying potential radiosensitization targets are urgently needed.

Spindlin1 (SPIN1), belonging to the SPIN/SSTY family, was originally described as a maternal transcript for mouse embryo development [5, 6]. Several studies have suggested that SPIN1 contributed to a plethora of physiological or pathological processes, including skeletal muscle development regulation, cardiomyocyte proliferation evaluation, cell cycle progression and chromosomal stability maintenance [7–9]. Increasing evidence has shown that human SPIN1 was frequently overexpressed in multiple malignant tumours, such as colorectal cancer, gastric cancer, glioma, and breast cancer [10–15]. Moreover, we previously demonstrated that SPIN1 promoted tumorigenesis and tumour progression by regulating the universal large ribosomal subunit protein 18 (uL18)–murine double minute 2 (MDM2)–p53 axis [16]. Chen et al. [17]. reported that LINC00473 facilitated radioresistance by regulating the miR-374a-5p/SPIN1 axis in oesophageal squamous cell carcinoma (ESCC). Yu et al. reported that cir_0001686 and circ_0007380 could decrease the radiosensitivity of ESCC cells by regulating the expression of miR-876-5p and miR-644a and downstream SPIN1 [18, 19]. However, how SPIN1 affects radioresistance in NSCLC cells is still unclear.

Forkhead box protein M1 (FOXM1), a member of the forkhead superfamily, is a crucial transcription factor of proteins involved in cell growth, invasion, metastasis and the DNA damage response (DDR) [20]. Previous studies have shown that FOXM1 was abundantly expressed in NSCLC and various other cancers [21, 22]. Moreover, FOXM1 overexpression was involved in regulating the cell cycle, apoptosis and DNA repair and thus can facilitate tumorigenesis and radioresistance via different mechanisms [21, 23–26]. FOXM1 protects cells from cytotoxic DNA damage via the upregulation of DNA repair proteins, including RAD51 and BRCA2 [27]. Previous studies have shown that FOXO3a binded to the promoter of FOXM1, thus impeding its transcription [20, 28]. More importantly, Im et al. demonstrated that FOXO3a/FOXM1-dependent DNA repair protected idiopathic pulmonary fibrosis (IPF) fibroblasts from radiation-induced cell death [26]. These studies strongly support that FOXM1 activation is tightly associated with oncogenesis and radioresistance.

In this study, we demonstrated that SPIN1 was a crucial factor in NSCLC radioresistance both in vitro and in vivo. Mechanistically, we revealed that SPIN1 promoted MDM2-mediated FOXO3a ubiquitination and subsequent FOXM1 activation. These results revealed a connection between SPIN1 and radioresistance and indicated that suppressing the activation of FOXM1 may be a feasible therapeutic strategy for radioresistant NSCLC.

## Results

### SPIN1 is highly expressed in NSCLC and predicts poor clinical outcomes in patients

**Fig. 1:**
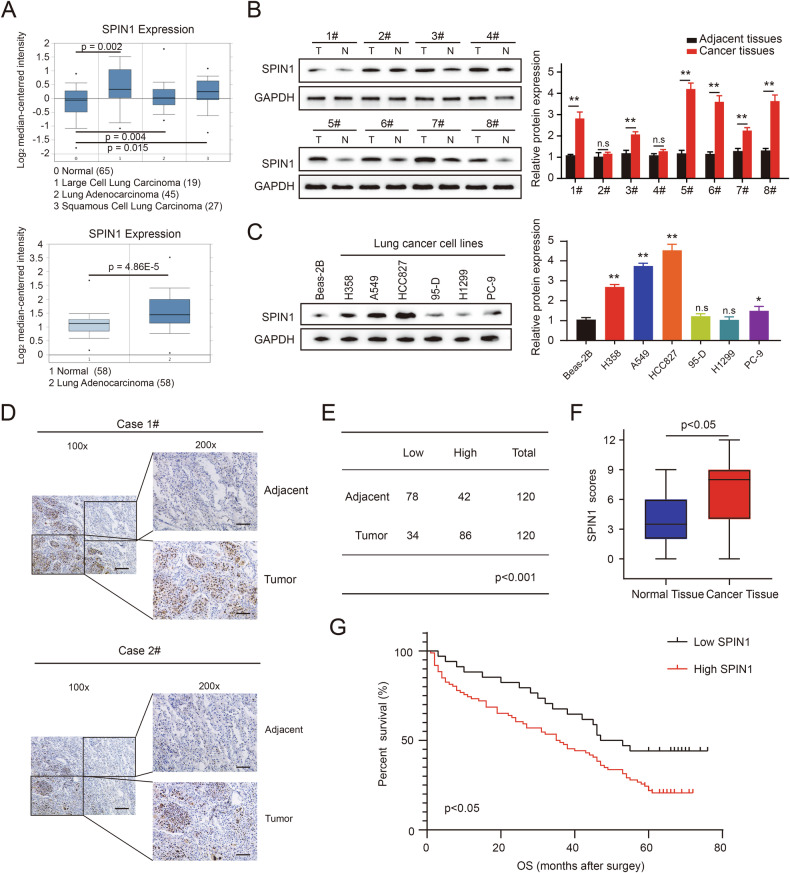
SPIN1 is highly expressed in NSCLC patients, and this phenotype predicts poor clinical outcomes. **A** The relative mRNA expression of SPIN1 in lung adenocarcinoma tissues compared with that in normal lung tissues obtained from the Oncomine database. **B** SPIN1 protein levels in fresh lung cancer tissues and paired normal tissues (*n* = 8) were analysed via western blotting. **C** The expression of SPIN1 in six NSCLC cell lines and normal bronchial epithelioid cells was detected by western blotting. **D** Representative images of SPIN1 immunohistochemistry in NSCLC tissues and adjacent nontumorous tissues (Left, scale bar: 100 μm; Right, scale bar: 50 μm). **E** Quantification of SPIN1 protein expression levels in NSCLC tissues and normal adjacent tissues. **F** IHC staining scores of the IHC images of NSCLC and adjacent nontumorous tissues. **G** Kaplan-Meier curves of patients with high and low SPIN1 expression in NSCLC (*n* = 120, log-rank test, *p* < 0.05). n.s., no significant difference, *p < 0.05, **p < 0.01. The cell experiments were conducted more than 3 times independently, and the data are presented as the means ± standard deviations (SDs).

Bioinformatics analysis was used to assess the SPIN1 mRNA expression level in NSCLC tissues. The results revealed that the mRNA expression of SPIN1 was upregulated in lung cancer tissues compared with normal lung tissues (Fig. 1A, https://www.oncomine.org/). Next, we quantified SPIN1 protein expression in 8 fresh NSCLC tissues and matched adjacent nontumor tissues, as well as 7 human lung cancer cell lines. As shown in Fig. 1B, C, the expression of SPIN1 was obviously elevated in lung cancer tissues and cell lines compared with normal tissues and Beas-2B cells, implying that SPIN1 is a crucial factor in NSCLC progression. In addition, IHC assays revealed that the level of SPIN1, located mainly in the nucleus, was greater in lung cancer tissues than in adjacent normal tissues (86/120, 71.6%, Fig. 1D, E). Similarly, the results of IHC staining further verified that SPIN1 expression was increased in NSCLC tissues (Fig. 1F). Notably, significant associations between SPIN1 expression and clinical TNM stage, tumour size, depth of invasion, and lymph node metastasis were detected in the analysis of paraffin-embedded lung adenocarcinoma tissues (Table 1, *p* < 0.05). Furthermore, high expression of SPIN1 was strongly correlated with poorer overall survival (OS) in NSCLC patients (Fig. 1G, *p* < 0.05). Taken together, these findings indicate that SPIN1 is upregulated in NSCLC and may play a crucial role in NSCLC progression.

**Table 1:** Relationships between SPIN1 expression and the clinical characteristics of NSCLC patients.

Parameters	*n* = 120	SPIN1 expression		*P* value
		Low (34)	High (86)	
Sex				
Male	73	22	51	0.585
Female	47	12	35	
Age (y)				
≤60	64	19	45	0.725
>60	56	15	41	
Differentiation				
Poor	70	21	49	0.632
Moderate/well	50	13	37	
Tumour size (cm)				
≤4	72	26	46	**0.021**
>4	48	8	40	
TNM stage				
I + II	61	24	37	**0.006**
III + IV	59	10	49	
T stage				
pT1 + pT2	67	25	42	**0.014**
pT3 + pT4	53	9	44	
Lymph node status				
N0	65	24	41	**0.023**
N1 + N2 + N3	55	10	45	
Total	120	34	86	

Bold values are statistically significant (*p* < 0.05).

### Knockdown of SPIN1 impairs NSCLC tumorigenesis both in vitro and in vivo

**Fig. 2:**
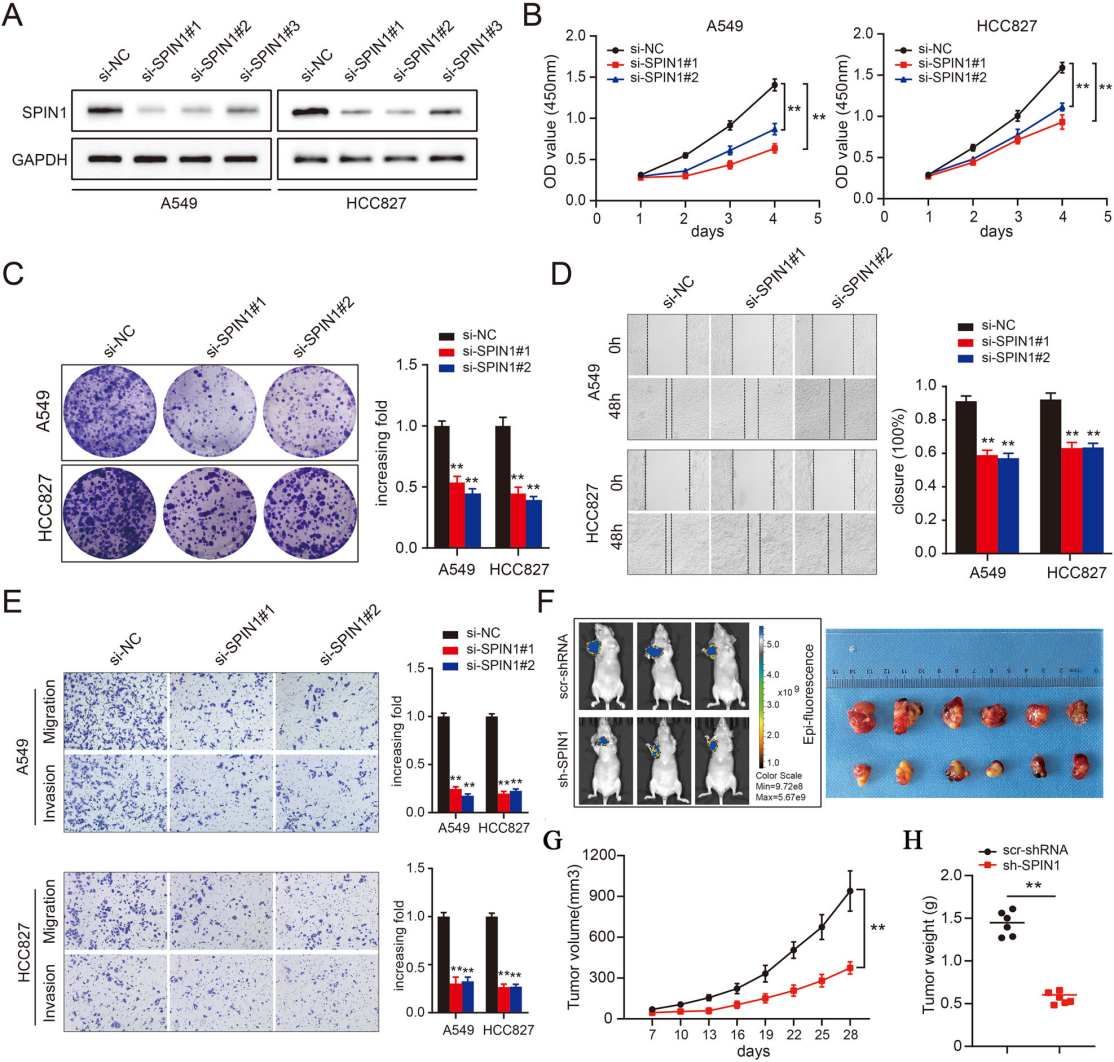
Knockdown of SPIN1 impaired tumorigenesis in NSCLC both in vitro and in vivo. **A** Western blotting assays were conducted to validate the transfection efficiency of SPIN1 siRNA in A549 (left) and HCC827 (right) cells. **B** CCK-8 assays were used to evaluate the growth ability of A549 and HCC827 cells transfected with SPIN1 siRNAs. **C** A colony formation assay was performed in SPIN1-depleted NSCLC cells. Scratch wound healing (**D**) and transwell (**E**) assays were conducted to evaluate the migratory and invasive abilities of NSCLC cells upon SPIN1 depletion. **F** Representative images of xenograft tumours in two groups (scramble groups and shSPIN1 groups). **G** Growth curves of xenograft tumours derived from A549 cells expressing scramble or SPIN1 shRNA are presented. **H** Histograms of tumour weights from the above experiments. **p < 0.01. At least three replicate experiments were performed, and the final results are presented as the means ± SDs.

Given its upregulation in NSCLC patients, we hypothesised that SPIN1 could promote tumorigenesis. SPIN1 expression was knocked down via siRNA silencing, and the high transfection efficiency was then verified by western blotting assays in A549 and HCC827 cells (Fig. 2A). As expected, depletion of SPIN1 inhibited the growth of lung cancer cells in the silenced group compared with that in the control group (Fig. 2B). Consistent with this notion, colony formation was also inhibited when SPIN1 was downregulated (Fig. 2C). We next assessed the migration and invasion abilities of NSCLC cells with SPIN1 knockdown. As shown in Fig. 2D, the depletion of SPIN1 decreased the wound healing rate, indicating that the loss of SPIN1 suppressed NSCLC cell migration. Consistently, the transwell assays provided additional compelling evidence that SPIN1 silencing inhibited cell migration and invasion (Fig. 2E). The function of SPIN1 was further evaluated in a nude mouse xenograft model in which SPIN1 was depleted via specific shRNAs. As outlined in Fig. 2F–H, the suppression of SPIN1 markedly inhibited tumour growth, as reflected by the tumour size, volume and weight. Taken together, these data verified the pro-oncogenic biological effects of SPIN1 and indicate that SPIN1 depletion impairs tumorigenesis in NSCLC both in vitro and in vivo.

### SPIN1 is associated with cell cycle redistribution and decreases radiation-induced DNA damage

**Fig. 3:**
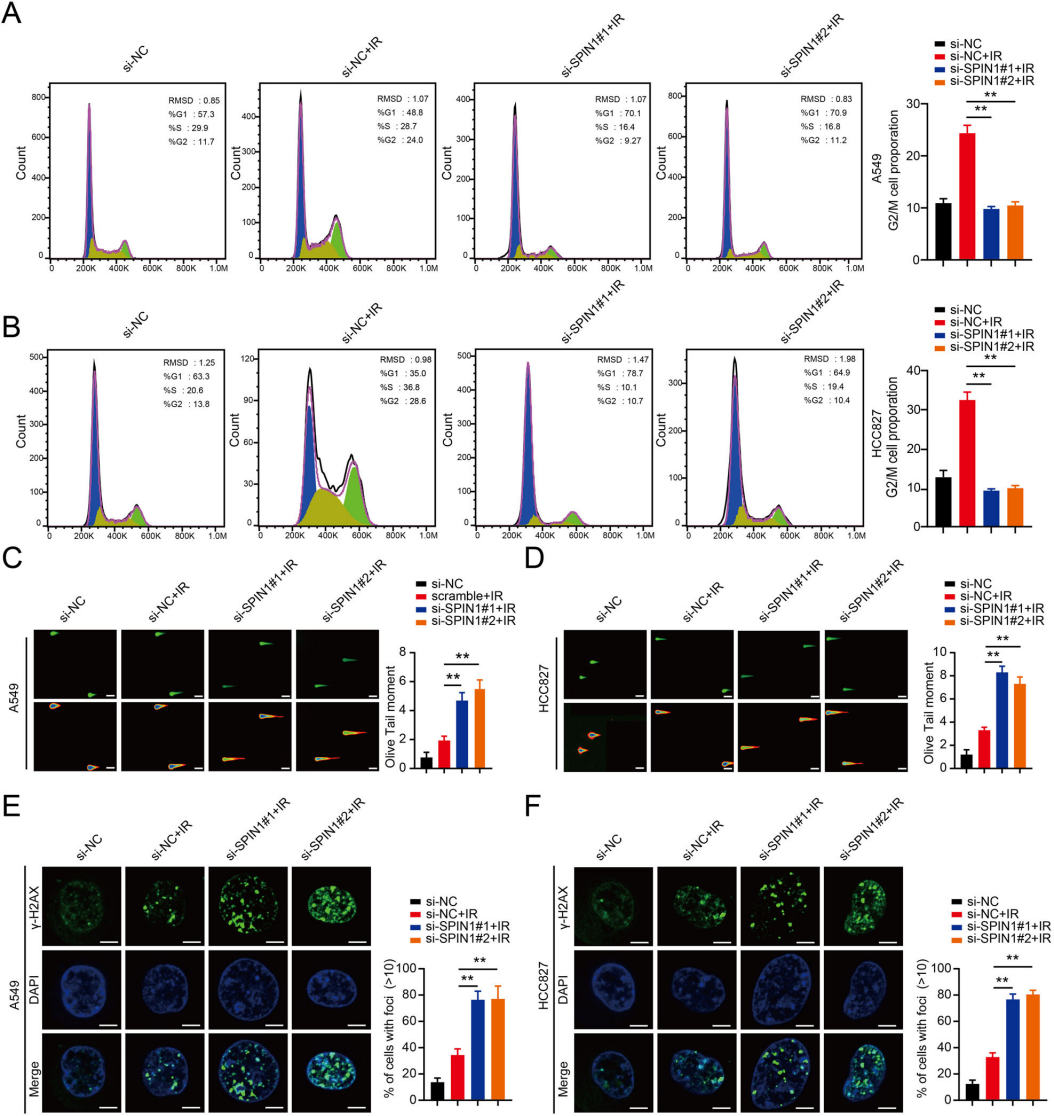
SPIN1 is associated with cell cycle redistribution and attenuates radiation-induced DNA damage. Representative images (**A**) and quantification analysis (**B**) of flow cytometry data depicting the cell cycle distribution of NSCLC cells (A549 and HCC827) transfected with NC and SPIN1 siRNAs 6 h after IR (6 Gy). **C**, **D** Representative images of neutral comet assays performed 4 h after IR exposure of SPIN1-depleted or control cells. Scale bar: 25 μm. **E**, **F** Immunofluorescence staining was performed to detect γ-H2AX foci formation in A549 and HCC827 cells transfected with SPIN1 siRNAs or negative control siRNAs. Scale bar: 10 μm. **p < 0.01. All the experiments were performed three times independently, and the results are presented as the means ± SDs.

Previous studies have shown that SPIN1 was essential for cell cycle redistribution and that SPIN1 downregulation sensitised cancer cells to DNA damage [9, 29]. Thus, we first assessed the role of SPIN1 in cell cycle redistribution and DNA damage repair in NSCLC cells exposed to ionising radiation (IR). As shown in Fig. 3A, B, a significant increase in the percentage of cells arrested in the G2/M phase was detected upon IR, and silencing of SPIN1 in both A549 and HCC827 cells resulted in marked decreased in the percentage of G2/M phase cells compared with that in the group treated with si-NC and IR (*p* < 0.01). These results suggest that SPIN1 is closely associated with cell cycle progression. To further clarify the role of SPIN1 in the DNA damage process, we conducted neutral comet assays upon irradiation in NSCLC cells. As presented in Fig. 3C, D, in addition to the cell DNA damage caused by radiation therapy, the depletion of SPIN1 also induced DNA damage. Compared with IR alone, SPIN1 knockdown combined with IR increased the olive tail moment (*p* < 0.01), indicating that IR induced a greater degree of DNA damage in the absence of SPIN1 in NSCLC cells.

Furthermore, we also performed γ-H2AX foci formation assays, as γ-H2AX foci are considered as critical indicators of DNA damage [30]. Consistent with the data in Fig. 3C, D, the formation of γ-H2AX foci occurred not only in the IR only group but also in the SPIN1 knockdown plus IR group. When both A549 and HCC827 cells were irradiated and SPIN1 was also knocked down, the number of γ-H2AX foci was significantly greater than that in the IR-only group (Fig. 3E, F). Collectively, these data support the notion that SPIN1 is associated with cell cycle regulation and attenuates cellular DNA damage.

### SPIN1 silencing increases radiosensitivity in vitro and in vivo

**Fig. 4:**
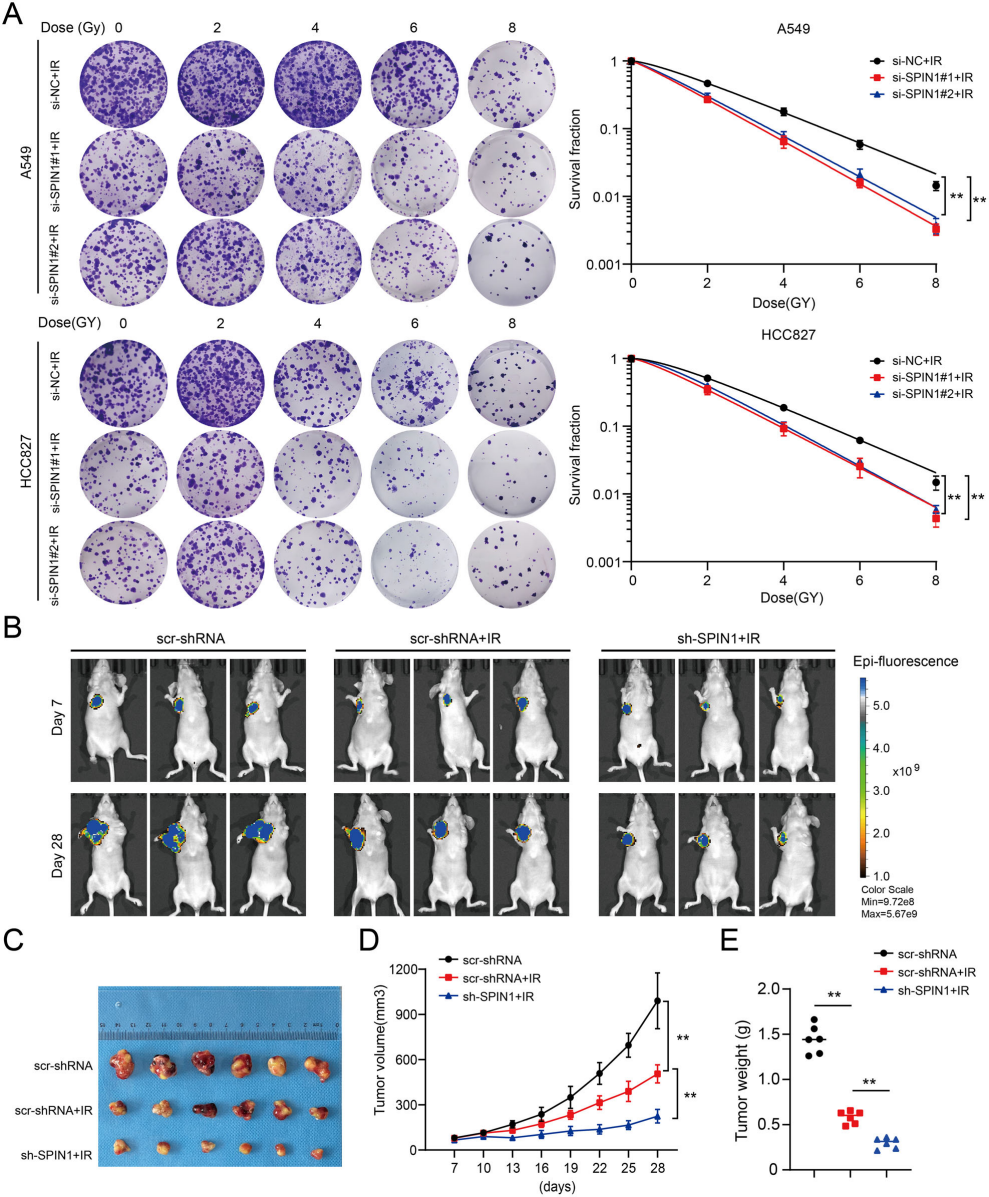
SPIN1 silencing increased radiosensitivity in vitro and in vivo. **A** clonogenic survival assay was performed to evaluate the radiation sensitivity of A549 and HCC827 cells transfected with SPIN1 siRNAs or control siRNA upon exposure to the indicated doses of IR 6 h later. **B**, **C** Representative images of xenograft tumours in the three indicated groups are shown. **D** Tumour volumes were calculated, and the data are presented as the geometric mean for each group versus time. **E** The weights of the tumours in the above three groups are presented. **p < 0.01.

Given the effects of SPIN1 on the cell cycle and IR-induced DNA damage, we speculated that SPIN1 is involved in radioresistance in NSCLC cells. To validate this hypothesis, we performed a clonogenic survival assay to assess cell survival upon irradiation. As shown in Fig. 4A, a decrease in SPIN1 increased the sensitivity of NSCLC cells to radiation. In addition, the role of SPIN1 in NSCLC radiosensitivity in vivo was assessed by using tumour xenograft models treated with or without irradiation. In line with the in vitro results, the tumour size and weight in the groups that underwent irradiation were significantly smaller than those in the control groups without irradiation, indicating that radiotherapy was effective. More interestingly, the tumour size and weight in the sh-SPIN1 groups exposed to irradiation were substantially smaller than those in the scramble groups with irradiation, suggesting that SPIN1 knockdown rendered NSCLC cells more susceptible to irradiation in vivo (Fig. 4B–E). Together, these results indicate that SPIN1 depletion increased radiosensitivity both in vitro and in vivo.

### SPIN1 is involved in the activation of FOXM1

**Fig. 5:**
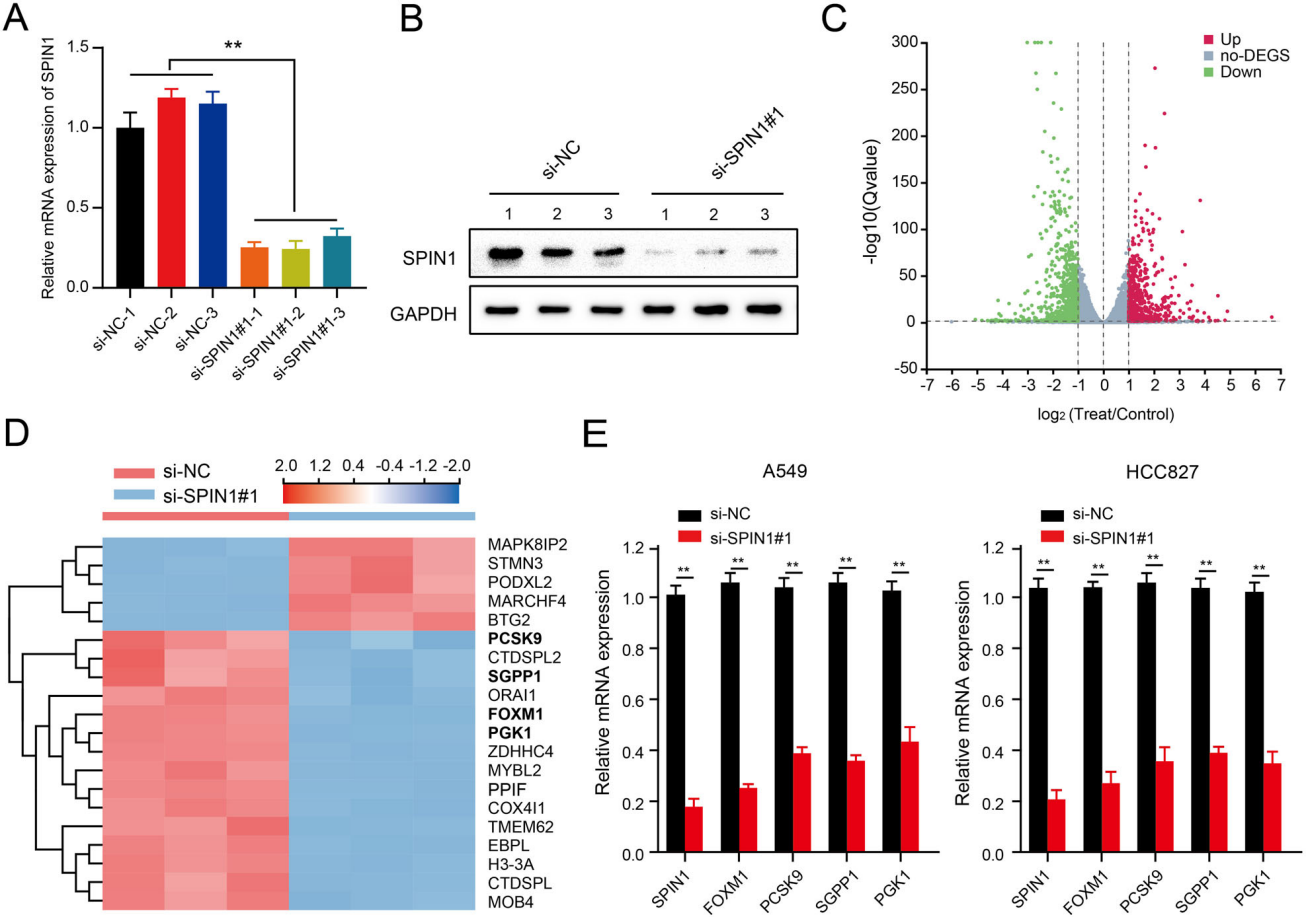
SPIN1 is involved in the activation of FOXM1. qRT‒PCR (**A**) and western blotting (**B**) were performed to determine the transfection efficiency of SPIN1 siRNA. **C** Volcano plot of the differentially expressed genes obtained from SPIN1-depleted or control cells. Red indicates the upregulated genes, whereas green represents the downregulated genes analysed from the RNA-sequencing data (fold change >3 and *p* < 0.01). **D** Heatmap of the changes in the top 20 representative mRNAs according to the RNA-seq data. **E** The mRNA levels of the indicated genes were determined via qRT‒PCR assays upon SPIN1 knockdown. **p < 0.01. The biological experiments were independently conducted 3 times. All the data are presented as the means ± SDs.

To elucidate the underlying downstream regulatory mechanisms mediated by SPIN1 in NSCLC, RNA sequencing (RNA-seq) was performed to compare the genomic expression profiles of A549 cells transfected with control or SPIN1 siRNAs. First, the transfection efficiency of the SPIN1 siRNAs was verified via western blotting and qRT‒PCR assays (Fig. 5A, B). As shown in Fig. 5C, 1422 significantly differentially expressed genes were identified between SPIN1-depleted A549 cells and control cells (*p* < 0.05 and fold change ≥2). The top 20 genes were selected on the basis of the magnitude of differential expression; among these genes, 5 genes were significantly upregulated, and the rest were downregulated (Fig. 5D). We selected 4 candidate genes (PCSK9, SGPP1, PGK1 and FOXM1) reported to be closely involved in tumour progression and radioresistance for further analysis [26, 31–33]. As shown in Fig. 5E, SPIN1 downregulation decreased the expression of PCSK9, SGPP1, PGK1 and FOXM1 in A549 and HCC827 cells, which was consistent with the RNA-seq data. Notably, among all these candidates, FOXM1 was the most downregulated gene upon SPIN1 depletion, therefore, we selected FOXM1 for further analysis.

### SPIN1 induces FOXM1 activation by promoting the ubiquitin-mediated degradation of FOXO3a

**Fig. 6:**
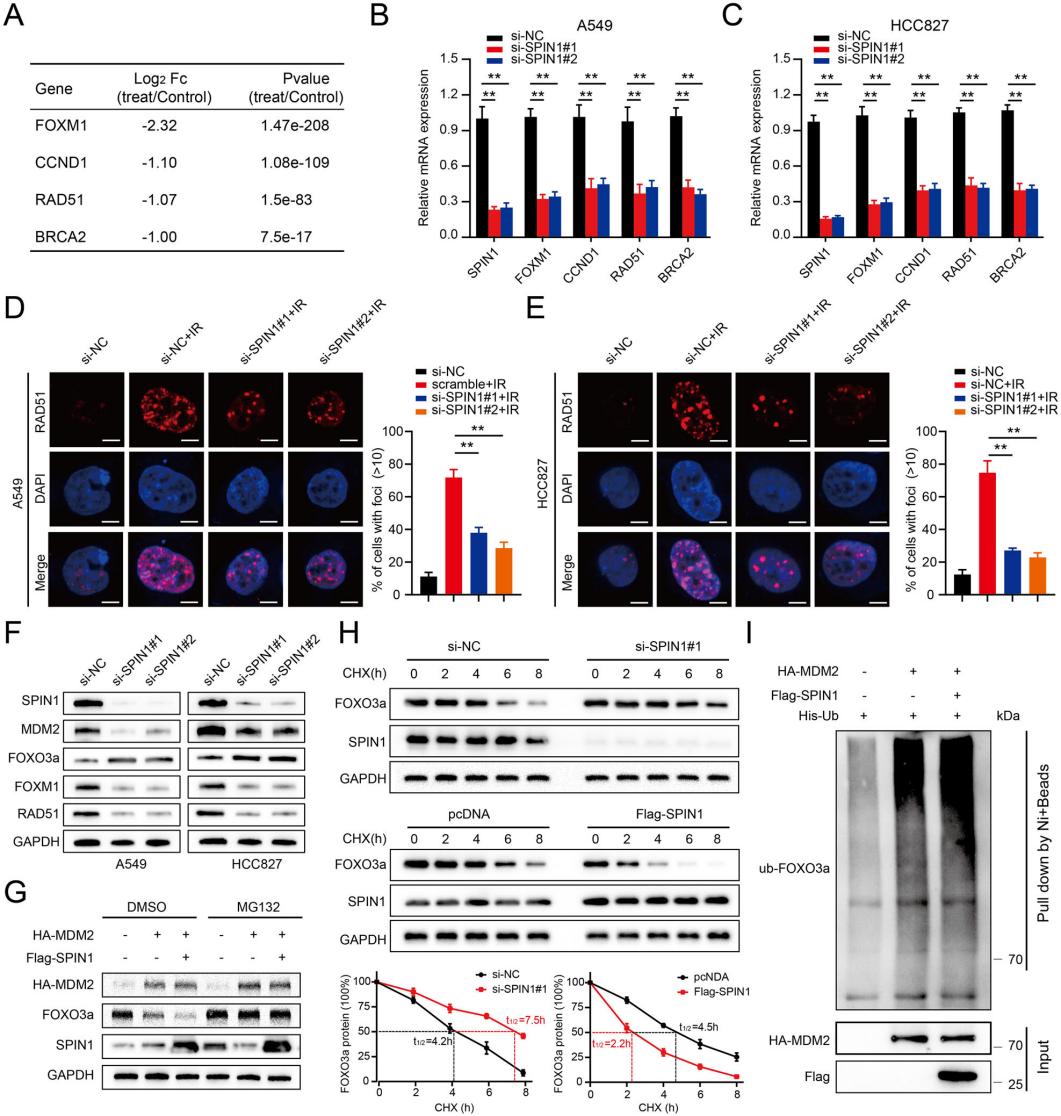
SPIN1 induces the activation of FOXM1 by promoting the ubiquitin-mediated degradation of FOXO3a. **A** List of several downstream targets of FOXM1 related to the cellular DDR (*p* < 0.05). **B**, **C** qRT‒PCR assays were utilised to validate the mRNA expression of the above genes upon SPIN1 knockdown in A549 and HCC827 cells. **D**, **E** Immunofluorescence staining showing the number of Rad51 foci in A549 and HCC827 cells after the indicated treatments (left panel, representative images; right panel, statistical data). Scale bar: 10 μm. **F** Western blotting assay showing the protein expression levels. **G** Western blotting was used to detect FOXO3a expression after pretreatment with MG132 for 6 h. **H** Western blot analysis of FOXO3a protein expression in modified HEK293T cells upon regulation of SPIN1 expression (upper panel). The line graph depicts the FOXO3a protein levels normalised to those of GAPDH at different time points (lower panel). **I** HEK293T cells were transfected with combinations of plasmids encoding His-Ub, HA-MDM2 or Flag-SPIN1 and incubated with MG132 for 6 h before being collected for in vivo ubiquitination analysis. **p < 0.01. At least three independent biological experiments were repeated, and the data are presented as the means ± SDs.

Several studies have shown that FOXM1 was involved in the regulation of the DDR in response to radiation-induced cell death [26]. To further validate whether SPIN1 confers radioresistance to NSCLC via the stabilisation of FOXM1, we first analysed the expression of FOXM1 and its relevant downstream molecules on the basis of the RNA-seq data (Fig. 6A). The qRT‒PCR results revealed that SPIN1 knockdown markedly inhibited the expression of FOXM1, CCND1, CCNB1, RAD51 and BRCA2, and all these factors were closely related to the DDR in A549 and HCC827 cells (Fig. 6B, C). RAD51 was identified as the key component in homologous recombination (HR) that was correlated with DNA damage repair [34]. Consistently, immunofluorescence staining assays revealed that DNA damage-induced RAD51 foci formation postirradiation was markedly impeded upon SPIN1 downregulation, indicating that SPIN1 depletion strongly impaired the DNA repair process in NSCLC cells exposed to irradiation (Fig. 6D, E).

Previous studies have demonstrated that FOXM1 was one of the most important downstream transcriptional targets of FOXO3a and plays a key role in the DNA damage response [27, 35]. More importantly, FOXO3a has been identified as a ubiquitinated substrate of MDM2 [36]. Furthermore, our study revealed that SPIN1 facilitates MDM2-mediated p53 ubiquitination and degradation [16]. Thus, we hypothesised that the MDM2-FOXO3a/FOXM1 axis might be involved in SPIN1-mediated radioresistance in NSCLC. As shown in Fig. 6F, the expression of FOXO3a was markedly increased, whereas the levels of FOXM1, MDM2 and RAD51 were significantly decreased in SPIN1-deficient cells, further suggesting that SPIN1 may be integral to the regulation of the FOXO3a/FOXM1 axis in NSCLC cells. To determine whether the SPIN1-mediated downregulation of FOXO3a protein expression was due to effects on FOXO3a stability, the half-life of the FOXO3a protein was assessed in treated HEK293T cells. As shown in Fig. 6H, SPIN1 knockdown markedly prolonged the half-life of FOXO3a from 4.2 h (in the control group) to 7.5 h. In contrast, SPIN1 overexpression greatly shortened the half-life of FOXO3a from 4.5 h to 2.2 h. Additionally, cotransfection of SPIN1 with MDM2 resulted in a greater decrease in FOXO3a protein expression, which was abrogated by MG132 treatment for 6 h (Fig. 6G). All of the above results indicate that SPIN1 decreases the expression of FOXO3a in an MDM2-dependent manner. More interestingly, the in vivo ubiquitination assays further indicated that the upregulation of SPIN1 markedly promoted the ubiquitination of the FOXO3a protein (Fig. 6I). Taken together, these results indicate that SPIN1 induces FOXM1 expression by promoting MDM2-mediated FOXO3a ubiquitination and degradation.

### FOXM1 is critical for SPIN1-mediated oncogenesis and radioresistance in NSCLC cells

**Fig. 7:**
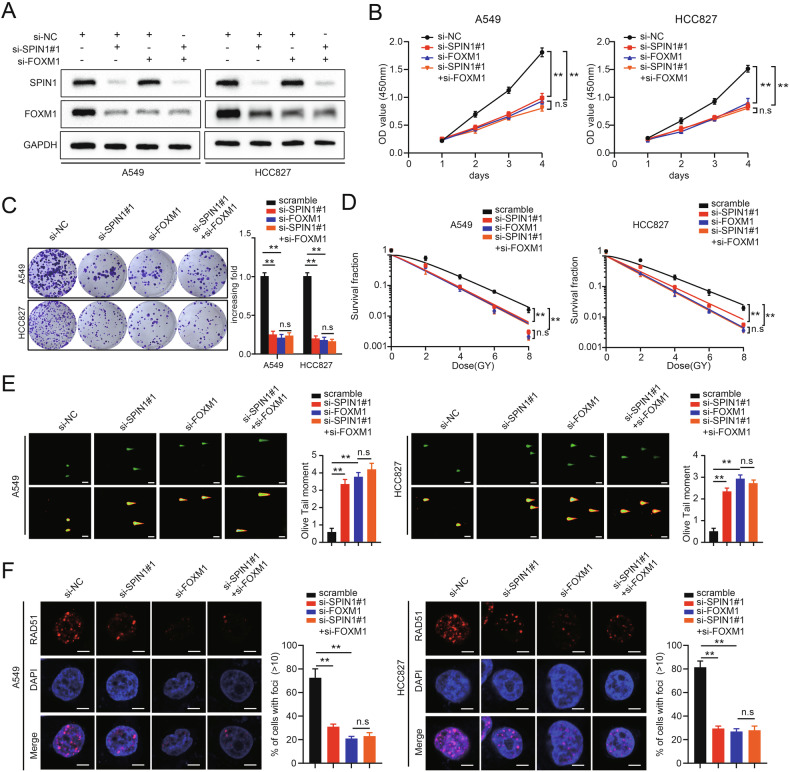
The attenuation of oncogenesis and radioresistance induced by SPIN1 depletion is mainly dependent on FOXM1 downregulation. **A** Cells (A549 and HCC827) transfected with siRNAs targeting SPIN1, FOXM1, or both were harvested and analysed via western blotting. **B** The proliferation and viability of the indicated cells were assessed via CCK-8 assays. Scale bar: 100 μm. **C** A colony formation assay was performed with the indicated cells. **D** Clonogenic cell survival assays were used to evaluate the radiation sensitivity of A549 and HCC827 cells transfected with the indicated siRNAs. **E** Neutral comet assays were conducted on the indicated transfected cells. Representative images (left panel) and quantification of the olive tail moment (right panel). Scale bar: 25 μm. **F** Immunofluorescence staining was performed to assess Rad51 foci formation. Scale bar: 10 μm. n.s., no significant difference, *p < 0.05, **p < 0.01. All experiments were performed independently at least three times, and the results are presented as the means ± SDs.

**Fig. 8:**
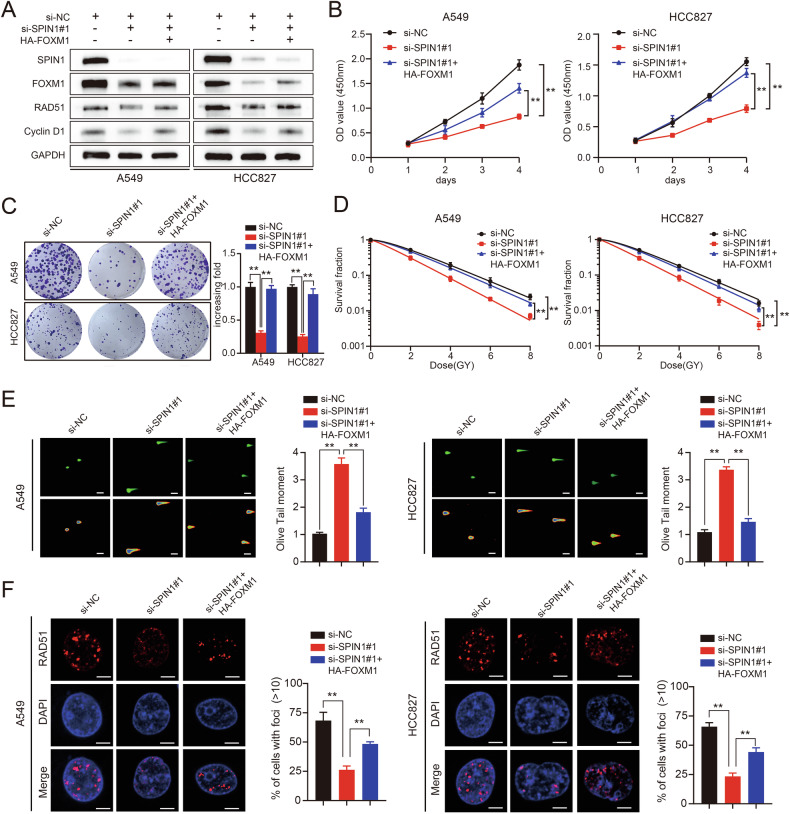
FOXM1 is critical for SPIN1-mediated oncogenesis and radioresistance in NSCLC cells. **A** Cells transfected with SPIN1 siRNAs and HA-FOXM1 plasmids were harvested, and the expression of relevant molecules was detected via western blotting. **B** The proliferation and viability of the indicated NSCLC cells were analysed via CCK-8 assays. Scale bar: 100 μm. **C** The results of the colony formation assay performed with the indicated cells. **D** A clonogenic survival assay was used to detect the sensitivity of the indicated cells (A549 and HCC827) transfected with SPIN1 siRNAs or/and FOXM1 plasmids. **E** The results of neutral comet assay performed in the three groups. Scale bar: 25 μm. **F** The number and number of Rad51 foci detected by immunofluorescence staining. Scale bar: 10 μm. n.s., no significant difference, *p < 0.05, **p < 0.01. All experiments were performed independently at least three times, and the results are presented as the means ± SDs.

**Fig. 9:**
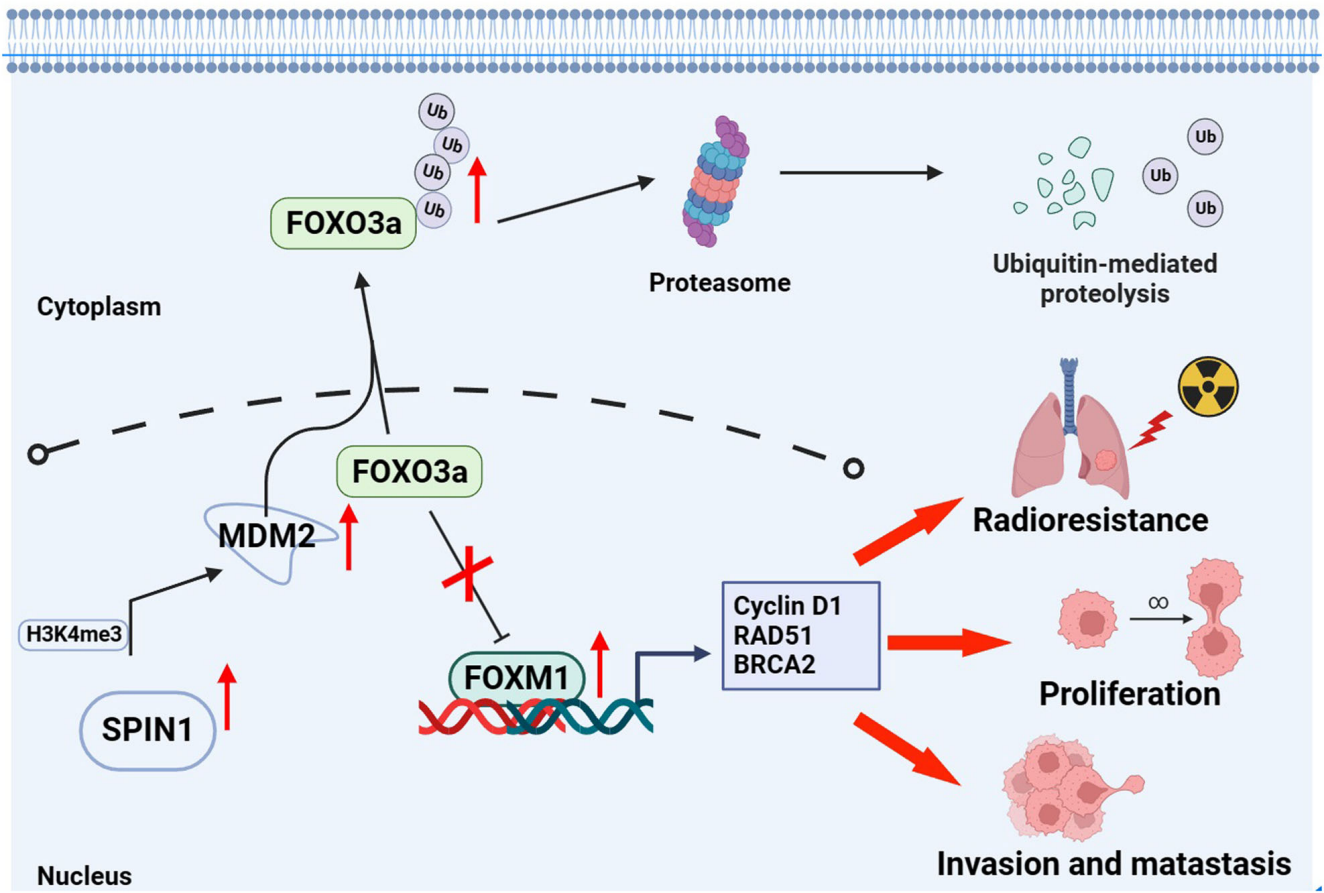
Molecular mechanism diagram. In this model, the overexpression of SPIN1 increases MDM2 expression by binding to H3K4me3 in its promoter region, inducing MDM2-mediated FOXO3a ubiquitination and degradation. The decrease in FOXO3a impairs its ability to suppress FOXM1 activity, thereby facilitating the transcription of downstream targets of FOXM1, including DNA damage repair genes, ultimately leading to NSCLC tumorigenesis and radioresistance.

To further clarify the role of FOXM1 in SPIN1-induced malignancy and radioresistance, rescue experiments were conducted. First, SPIN1 siRNA was transfected into cells with or without FOXM1 depletion (Fig. 7A). As shown in Fig. 7B–D, FOXM1 depletion markedly inhibited cell growth and proliferation, further clarifying the functions of FOXM1 in tumorigenesis. More intriguingly, our data revealed that SPIN1 depletion had no statistically significant effect on these phenotypes when FOXM1 was simultaneously knocked down (Fig. 7B–D). In addition, we found that silencing FOXM1 increased the radiosensitivity of NSCLC cells, as reflected by the increase in the DNA damage level and decreased number of Rad51 foci (Fig. 7E, F). Consistently, further inhibition of SPIN1 had little effect on the radiosensitivity of NSCLC cells cotransfected with FOXM1 siRNA (Fig. 7E, F). These findings demonstrate that SPIN1 drives NSCLC carcinogenesis and radioresistance in a FOXM1-dependent manner. To further determine the role of FOXM1 in SPIN1-induced malignancy and radioresistance, the following rescue experiments were conducted. The exogenously expressed FOXM1 plasmid was transfected into SPIN1-depleted cells. As expected, the downregulation of FOXM1, RAD51 and cyclin D1 expression caused by SPIN1 depletion was partly reversed by FOXM1 upregulation (Fig. 8A). Additionally, the suppression of cell growth and proliferation induced by SPIN1 knockdown was partially restored by FOXM1 overexpression (Fig. 8B–D). Similarly, the upregulation of FOXM1 markedly attenuated the increases in DNA damage and radiosensitivity and the decrease in IR-induced DNA repair induced by SPIN1 depletion (Fig. 8E, F). Taken together, the results of our study support that FOXM1 is responsible for SPIN1-mediated oncogenesis and radioresistance in NSCLC cells. On the basis of the aforementioned data and our previous study, we propose a working model for the mechanisms of SPIN1 in NSCLC radioresistance (summarised in Fig. 9).

## Discussion

Lung cancer is the deadliest malignancy worldwide, despite the development of numerous therapeutic methods [37]. Radiotherapy plays essential roles in different stages of lung cancer, and radioresistance limits the efficacy of radiotherapy [38]. Thus, in-depth investigations of the underlying mechanisms of radioresistance in NSCLC are crucial for the development of effective anticancer therapies. In this study, we determined the biological role of SPIN1 in promoting NSCLC progression and radioresistance in vitro and in vivo. In addition, we demonstrate for the first time that SPIN1 regulates FOXO3a/FOXM1 axis by modulating MDM2 activity, thereby promoting oncogenesis and radioresistance in NSCLC.

Our study provides a better understand of the role of SPIN1 in NSCLC and revealed that SPIN1 is highly expressed and correlated with aggressive behaviours and poor prognosis in NSCLC patients. Functional studies further revealed that the loss of SPIN1 suppressed cell proliferation, cell cycle progression and invasion both in vitro and in vivo. Previous studies have shown that cell DNA double-strand breaks (DSBs) occur when nonhaematopoietic cells are exposed to a high dose of radiation [39]. Subsequently, HR-mediated repair is normally initiated with the recruitment of the repair protein RAD51 by breast cancer-associated gene 2 (BRCA2) to damaged DNA sites, which is the main mechanism of DNA repair that limits the effects of radiation [40, 41]. Notably, a recent study revealed that circ_0086720 downregulation increased the sensitivity of NSCLC cells to radiation by targeting the miR-375/SPIN1 axis, indicating that SPIN1 may serve as a radioresistance-promoting protein in NSCLC [42]. Although several evidence suggests that SPIN1 could be a predictive marker of chemoradioresistance, whether SPIN1 plays a critical role in the resistance of NSCLC to IR, which also induces DNA damage, is still largely unclear. Our experiments revealed for the first time that depletion of SPIN1 increased the DNA damage level and suppressed DNA repair processes, ultimately rendering NSCLC cells and xenograft tumours more susceptible to irradiation. These results suggest that increased SPIN1 expression was closely associated with tumorigenesis and radioresistance and that targeting SPIN1 might be a therapeutic strategy for sensitising NSCLC cells to radiotherapy.

Aberrant dysregulation of the FOXO3a/FOXM1 axis contributes greatly to carcinogenesis and chemoradioresistance in multiple cancers [27, 36, 43]. However, whether the FOXO3a/FOXM1 axis is involved in the malignancy and radioresistance of NSCLC is unclear. Our study revealed for the first time the association between SPIN1 and the FOXO3a/FOXM1 axis in NSCLC cells. RNA-seq, western blotting and qRT‒PCR assays revealed that silencing SPIN1 led to a reduction in FOXM1 expression at both the transcriptional and posttranscriptional levels. Given that MDM2 is the predominant negative regulator of FOXO3a [36] and that SPIN1 binds to and retains uL18 in the nucleolus, releasing MDM2 and restoring its E3 ubiquitin ligase activity [16], we speculated that SPIN1 might regulate the MDM2‒FOXO3a‒FOXM1 axis in NSCLC cells. As expected, our mechanistic analysis indicated that SPIN1 promotes MDM2-mediated FOXO3a ubiquitination and degradation, thereby increasing FOXM1 protein expression. Notably, the work of Lv et al. indicated that SPIN1 induces MDM2 expression by binding to H3K4me3 of the MDM2 promoter region, thereby activating the MDM2-p21-E2F1 pathway to promote gastric cancer cell proliferation [12]. Hence, SPIN1 may also reinforce MDM2 activity via similar mechanisms to modulate the FOXO3a/FOXM1 axis, which needs to be investigated in our future studies.

Furthermore, we performed reverse validation experiments. These results further revealed that the biological effects of SPIN1 depletion on the proliferation and radioresistance of NSCLC cells could be partly restored by FOXM1 overexpression. These results strongly suggest that FOXM1 is a major but not the only downstream effector of SPIN1 in NSCLC cells. An increasing number of studies have demonstrated that the Wnt/β-catenin, PI3K/Akt, and p53 pathways participate in SPIN1-mediated aggressive behaviours and therapy resistance [10, 14, 16]. Thus, we suspect that these cancer-related pathways may also contribute to SPIN1-induced NSCLC progression and radioresistance.

Overall, our study revealed a novel, potentially targetable mechanism: SPIN1 promotes the MDM2-mediated ubiquitination and degradation of FOXO3a, inducing FOXM1 expression and eventually leading to NSCLC progression and radioresistance.

## Conclusion

Taken together, our findings not only elucidated the molecular mechanism by which FOXO3a/FOXM1 signalling is modulated by SPIN1 but also indicate that targeting SPIN1 might serve as a potential radiosensitizer for NSCLC intervention.

## Materials and methods

### Patients and sample collection

A total of 120 NSCLC tissues and corresponding normal tissues were acquired at the First Affiliated Hospital of Nanchang University from January 2014 to September 2018. All the patients enroled in our study were diagnosed with primary NSCLC and underwent surgery without any systemic or local therapy. The clinical information of the patients and the statistical information are summarised in Table 1. The detailed information of the patients used in our experiments is presented in Supplement 1. Cancerous and paracancerous tissues from eight NSCLC patients were obtained from January 2014 to July 2016. All patient-related research was authorised by the First Affiliated Hospital of Nanchang University Ethics Committee.

### Immunohistochemistry (IHC)

The immunohistochemical staining assay was performed according to the standard methods we described previously [10, 44]. In brief, the paraffinized sections were deparaffinized and hydrated in sodium citrate buffer for antigen retrieval and subsequently incubated with a primary antibody against SPIN1 (1:100, 19531-1-AP, Proteintech) overnight at 4 °C. On the second day, the sections were stained with 3,3’-diaminobenzidine (DAB) and counterstained with haematoxylin upon incubation at room temperature with secondary antibodies (1:1000, ab288151, Abcam). The staining of deparaffinized slices were independently evaluated and scored by two blinded, experienced pathologists in accordance with the staining index criteria used in our previous study [45].

### Human cell lines and cell culture

Human NSCLC cell lines (95-D, A549, HCC827, H358, H1299 and PC-9), as well as normal human bronchial epithelioid cells (Beas-2B), were purchased from Procell Life Science and Technology (Wuhan, China) and cultured in Roswell Park Memorial Institute 1640 (RPMI-1640) medium. The HEK293T cells used in our work were kindly provided by Professor Pan (Nanchang University) and incubated in Dulbecco’s modified essential medium (DMEM). All the cells were cultured in the indicated media supplemented with 10% foetal bovine serum (FBS, Biological, Kibbutz Beit Haemek, Israel) and 1% penicillin/streptomycin at 37 °C with 5% CO_2_.

### Cell transfection

The HA-FOXM1 plasmid, SPIN1 siRNAs and their negative control pcDNA/siRNAs were purchased from GenePharma (Shanghai, China). The Flag-SPIN1, His-ubiquitin and HA-MDM2 plasmids were kind gifts from Professor Hua Lu (School of Medicine, Tulane University). The following sequences of the siRNAs were used: SPIN1 siRNA#1, 5′-GCAUUAUGCCUGAUUCCAATT-3′; SPIN1 siRNA#2, 5′-GGUCCGAGCAAACCUGUUUTT-3′ [10]; and FOXM1 siRNA 5′-CUCUUCUCCCUCAGAUAUATT-3′. All the above constructs were transfected into the indicated cells at 50–70% cell density via TurboFect transfection reagent (Thermo Scientific, USA) according to the manufacturer’s instructions. Subsequent experiments were carried out at 36–48 h post-transfection.

### Western blotting assay

Western blotting analysis was performed as previously described [46]. The indicated cell lysates extracted from fresh NSCLC tissues and xenograft tumours were prepared in RIPA buffer (APPLYGEN, China) with 1% protease/phosphatase inhibitor, denatured at 100 °C for 10 min and separated on 6% or 10% SDS‒PAGE gels. The proteins transferred from polyvinylidene difluoride (PVDF) membranes (Bio-Rad) were incubated with the indicated primary antibodies overnight, followed by incubation with the appropriate secondary antibodies the next day. The protein bands on the membranes were subsequently detected with an enhanced chemiluminescence kit (Thermo Scientific, USA). The primary antibodies used were as follows: SPIN1 (1:1000, 12105-1-AP, Proteintech), FOXM1 (1:1000, sc-37647, SANTA), FOXO3a (1:1000, 66428-1-1 g, Proteintech), RAD51 (1:1000, ab63801, Abcam), cyclin D1 (1:1000, 60186-1-1 g, Proteintech), MDM2 (1:800, 86934, Cell Signalling Technology), ubiquitin (1:1000, 10201-2-Ap, Proteintech), β-actin (1:10000, 66009-1-Ig, Proteintech) and GAPDH (1:15000, 60004-1-Ig, Proteintech). And all the full and uncropped western blots are summarised in Supplement 2-4.

### RNA transcriptome sequencing assay

HCC827 cells transfected with SPIN1 siRNAs or negative controls for 48 h were used for RNA sequencing (RNA-seq). Total RNA was extracted from HCC827 cells via TRIzol reagent (Takara, Japan), and RNA integrity was further validated. The RNA sequencing process was performed with the MGI2000 platform (MGI-Wuhan). Differentially expressed genes that met the established criteria of a false discovery rate (FDR) of <5% and a fold change of >2.0 were selected for further analysis. The RNA-seq data have been deposited in the Gene Expression Omnibus database under accession number GSE276500 (https://www.ncbi.nlm.nih.gov/geo/query/acc.cgi?acc=GSE276500).

### Quantitative real-time PCR (qRT‒PCR) assay

TRIzol reagent (Takara, Japan) was used to extract RNA from the experimental cells according to the manufacturer’s instructions as previously described [16]. First-strand cDNA was subsequently synthesised using the PrimeScript RT Reagent Kit (Takara, RR047A), and mRNA expression was determined via the SYBR® Premix Ex Taq™ Kit (Takara, RR420A). GAPDH was used as an internal control. The experiment was repeated in triplicate, and the 2^-ΔΔCt^ method was used to calculate the relative gene expression. The sequences of the primers utilised in our study are summarised in Table 2.

**Table 2:** Primer information.

Gene	Forward primer	Reverse Primer
SPIN1	CGGGATCCAAGACCCCATTCGGAAAGACA	CCGCTCGAGCTAGGATGTTTTCACCAAATCGTA
PCSK9	GGAACCTGGAGCGGATTACC	TTTCCCGGTGGTCACTCTGT
SGPP1	ATGGTCCTCCTCACCTATGG	TCAATCAGGTCCACAAATGG
PGK1	AACCAGAGGATTAAGGCTGC	GCCTACACAGTCCTTCAAGA
FOXM1	GGAGGAAATGCCACACTTAGCG	TAGGACTTCTTGGGTCTTGGGGTG
CCND1	CCTCTTCACCTTATTCATGGCTGA	CGTATCGTAGGAGTGGGACAGGT
CCNB1	TTTAGGGTGGGCAAGTCAGC	ATAAACCAGGCATTTGCAGGC
RAD51	CAACCCATTTCACGGTTAGAGC	TTCTTTGGCGCATAGGCAACA
BRCA2	TGCGTTGAGGAACTTGTGACTA	TGCGTTGAGGAACTTGTGACTA
GAPDH	CTTTGGTATCGTGGAAGGACTC	GTAGAGGCAGGGATGATGTTCT

### Cell Counting Kit-8 (CCK-8) assay

Briefly, the transfected cells were plated in a 96-well plate at a density of 2 × 10^3^ cells/well. Then, 10 µL of CCK-8 solution (Glpbio, USA) with 100 µL of complete medium was added to each well, and the cells were incubated for 30 min. The proliferation ability was measured at an absorbance of 450 nm for four consecutive days.

### Colony formation assay

Upon transfection for 48 h, the indicated cells were plated in 6-well plates and cultured in fresh medium for nearly two weeks. Then, the colonies were fixed and stained in 4% paraformaldehyde with 5% crystal violet for 20 min. Finally, the surviving colonies containing more than 50 cells were counted.

### Scratch wound assay

NSCLC cells transfected with the indicated components were plated in a 6-well plate and allowed to grow until full confluency. A 20 μL sterilised pipette tip was then used to draw straight lines to make wounds. Images of each well were taken at different times for further comparison, as previously described [47].

### Transwell assay

Transwell assays were performed as described previously [45]. The indicated NSCLC cells were resuspended in serum-free medium and plated in the upper chamber (Corning, USA), which was coated with 60 µL of Matrigel (Corning, USA) for the invasion assay. Matrigel was not used for the migration assay. After incubation for 48 h, the invading or migrating cells on the bottom surface of the upper chamber were fixed, stained and counted.

### Clonogenic cell survival assay

The cells transfected with the indicated siRNAs were collected and seeded in triplicate into 6-well plates at a certain density gradient and exposed to irradiation at the indicated doses ranging from 0, 2, 4, 6 and 8 Gy using an X-ray irradiator (Varian, USA). Two weeks later, the cell colonies containing more than 50 cells were photographed and counted under a microscop.

### Cycloheximide (CHX) chase assay

The stability of the FOXO3a protein was analysed via the CHX assay as previously described [48]. The indicated cells were collected and lysed at different time points upon the addition of 100 μg/mL CHX. The abovementioned cells were lysed, and the proteins were subjected to western blot analysis.

### Flow cytometry analysis

The indicated NSCLC cells were exposed to 6 Gy irradiation and then collected for cell cycle analysis. The samples were then stained with 2 mg/mL propidium iodide (PI; Roche, Switzerland) and 10 mg/mL RNase (Takara, Japan) for 30 min and subsequently analysed via flow cytometry (Mindray, China).

### Neutral comet assay

The neutral comet assay was performed three times via a Trevigen comet assay kit (C2041M, Beyotime, China) according to the manufacturer’s instructions. Briefly, the transfected cells were exposed to 6 Gy ionising radiation (IR) and harvested for 6 h. The cells were immobilised on comet slides with low-melting agarose, lysed for 1 h and washed with neutral electrophoresis buffer. The cells were subsequently subjected to electrophoresis at 21 V for 1 h at 4 °C. The gels were then neutralised and stained with SYBR^TM^ Gold (S11494, Invitrogen, USA) and photographed using a fluorescence microscope. CometScore 2.0 and GraphPad Prism software were used to analyse the olive tail moments of each group.

### Immunofluorescence staining

The transfected cells irradiated at 6 Gy were grown on confocal dishes. The cells were subsequently fixed with cold methanol and permeabilized with 0.5% Triton X-100 or cold acetone for 30 min. Five percent bovine serum albumin was used to block the cells at room temperature for 30 min. The cells were subsequently incubated with γ-H2AX (1:200, ab22551, Abcam) or Rad51 antibodies (1:200, ab133534, Abcam) overnight at 4 °C. After incubation with the corresponding secondary antibodies for 30 min, the cells were counterstained with 4’-6-diamidino-2-phenylindole (DAPI) and examined with a confocal fluorescence microscope (ZEISS, Germany).

### In vivo xenograft mouse model experiments

The mouse experiments were performed as previously described [45]. Briefly, 5-week-old female BALB/c mice were purchased from Hangzhou Ziyuan Laboratory Animal Technology Company (China). For tumorigenesis analysis, the mice were randomly divided into two groups (scr-shRNA and sh-SPIN1), and 1 × 10^7^ A549 cells were subcutaneously injected into the left axillary region. For the radioresistance assays, the mice were randomly classified into three groups (scr-shRNA, scr-shRNA + IR, and sh-SPIN1 + IR). The tumours were subjected to 2 Gy local irradiation for 5 consecutive days when the xenograft tumours reached a calculated average volume of 100 mm^3^ [4]. Tumours were measured (using callipers) and weighed every three days. The tumour volume was calculated via the following formula: volume = (length × width^2^)/2 (mm^3^). These experiments in xenograft mouse models were approved by the Ethics Committee of the First Affiliated Hospital of Nanchang University.

### Ubiquitination assay in vivo

This assay was performed as described previously [16]. HEK293T cells were plated in 10-cm dishes at 30% density and transfected with plasmids encoding Flag-SPIN1, His-Ub, FOXO3a or HA-MDM2 as indicated in the figure legends for 36 h. The cells were harvested and lysed upon incubation with the proteasome inhibitor MG132 (10 μM) for 6 h. The samples were subjected to western blotting analysis after being lysed in NETN buffer containing His-magnetic beads (MBL, China) overnight. Eluted proteins were detected by western blotting assays with the indicated antibodies.

### Statistical analysis

All the data are presented as the means ± SDs of a minimum of three biological replicates and were analysed via SPSS software version 26.0 (SPSS Inc., Chicago, IL). The differences between two groups were assessed via two-tailed Student’s *t* test. For multiple comparisons, ANOVA with a *post hoc* test was performed. The χ^2^ test was used to assess the correlations between SPIN1 expression and clinicopathological parameters. The log-rank test was used to analyse the survival data of NSCLC patients. Differences with *p* < 0.05 were considered statistically significant.

## Supplementary information

Supplementary 1

Supplementary 2

Supplementary 3

Supplementary 4

## Data Availability

The datasets used and/or analysed during the current study are available from the corresponding author upon reasonable request.
