# Supplementary figures and images for: SPIN1 accelerates tumorigenesis and confers radioresistance in non-small cell lung cancer by orchestrating the FOXO3a/FOXM1 axis
Source: Cell Death Dis. 2024 Nov 15;15(11):832. doi: 10.1038/s41419-024-07225-0 (PMC11568276; doi:10.1038/s41419-024-07225-0)

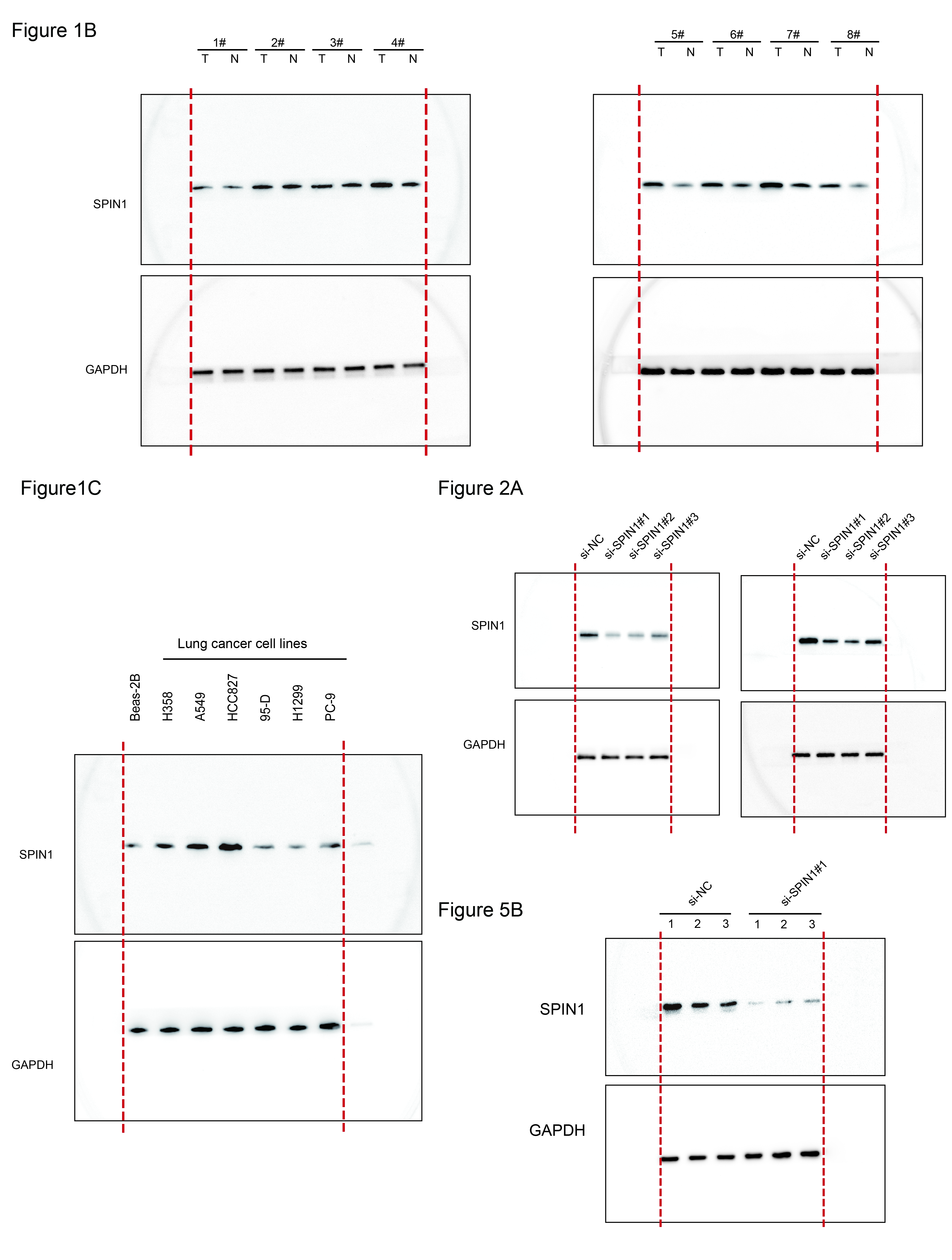

Supplement: Supplementary file 2 — Supplementary 2 [file 41419_2024_7225_MOESM2_ESM.tif]

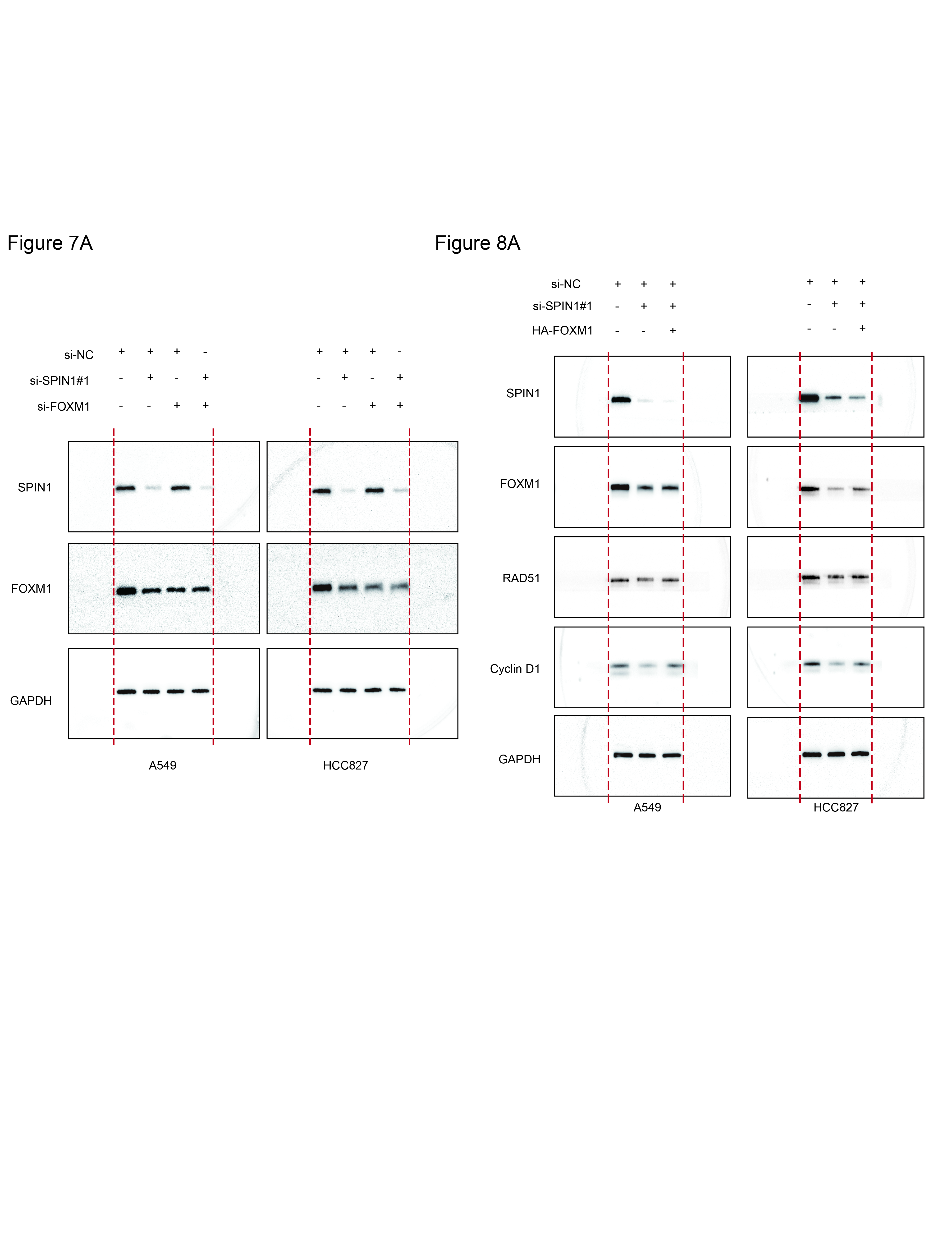

Supplement: Supplementary file 4 — Supplementary 4 [file 41419_2024_7225_MOESM4_ESM.tif]
